# Innate and Acquired Cellular Immunity in Children with Familial Hypercholesterolemia Treated with Simvastatin

**DOI:** 10.3390/jcm11102924

**Published:** 2022-05-22

**Authors:** Radosław Motkowski, Marek Alifier, Paweł Abramowicz, Jerzy Konstantynowicz, Bożena Mikołuć, Anna Stasiak-Barmuta

**Affiliations:** 1 Department of Pediatrics, Rheumatology, Immunology and Metabolic Bone Diseases, Medical University of Bialystok, 15-274 Bialystok, Poland; pawel.abramowicz@umb.edu.pl (P.A.); jurekonstant@o2.pl (J.K.); bozena.mikoluc@umb.edu.pl (B.M.); 2 Department of Clinical Immunology, Medical University of Bialystok, 15-274 Bialystok, Poland; anna.stasiak-barmuta@umb.edu.pl

**Keywords:** atherosclerosis, statins, flow cytometry, adhesion molecules, children, familial hypercholesterolemia, TLRs

## Abstract

The aim of this cross-sectional study was to assess the influence of simvastatin treatment in children with familial hypercholesterolemia (FH) on parameters of cellular immunity. Twenty-six children with FH were included, of which thirteen were treated with 10 mg simvastatin for at least 26 weeks, and thirteen were age- and sex-matched with a low-cholesterol diet only. Total WBC count and lipid profile were measured. Flow cytometry was used to identify lymphocyte subsets and determine the expression of adhesion molecules (AM) and toll-like receptors (TLRs) on leukocytes. No differences were found in the basic values of peripheral blood count and subpopulations of lymphocytes between groups. The percentage of granulocytes with the expression of AM was higher in those treated with statins. The TLR-2 expression on granulocytes and monocytes showed higher values, whereas the TLR-4 expression was lower on lymphocytes and granulocytes in simvastatin-treated children. Treatment with simvastatin in children with FH is not associated with alterations in the amounts of granulocytes and monocytes. There is no association between statin treatment and the pattern of peripheral blood lymphocyte subpopulations. The role of AM and TLRs needs further investigation, given the effect of statins on the innate immunity may be important for their efficacy and safety during growth.

## 1. Introduction

Familial hypercholesterolemia (FH) is a congenital metabolic disorder associated with mutations in different genes (OMIM #143890, #144010, #107703, #607786) that leads to an increase in serum low-density lipoprotein cholesterol (LDL-C), premature atherosclerosis and cardiovascular diseases. The prevalence of heterozygous FH in Europe has been estimated from 1 in 200 to 1 in 250 according to recent studies [1]: homozygous 1 in 1,000,000 [2]. Cardiovascular mortality in these patients is 100 times higher compared with the general population [3,4], whereas early implementation of hypolipemic treatment may significantly alleviate CVD risk later in life [2,5].

According to current clinical practice, the most commonly used hypolipemic drugs are statins. The safety of their use in adults has been evaluated in randomized placebo-controlled trials [6], while the data on the use of statins in children are still very limited. A recent meta-analysis of the “Cochrane Library” of available reports covering a total of 1177 children has shown that statins may reduce LDL cholesterol concentrations in children by an average of 30% (ranging from 23% to 40%) [7]. The studies included in the meta-analysis have assessed the safety profile of statins in a pediatric population; however, this is only to the extent of adverse events previously reported in adults, i.e., muscle pain, increased aminotransferase and creatinine kinase activities and rhabdomyolysis, also including the potential effect on height trajectory, weight gain and puberty.

Currently, atherosclerosis is defined as a chronic inflammatory disease resulting in atherosclerotic plaque formation. Thus, cytokines, adhesion molecules and receptor proteins produced by stimulated cells, as well as the immune system cells per se, are important biomarkers of coronary disease risk [8].

The first stage of immune response is innate immunity formed by phagocytes and antigen-presenting cells. The interactions between these cells depend on the expression of adhesion molecules and toll-like receptors (TLRs). Among the adhesion molecules found in the cells involved in the congenital response, the CD11a molecule plays a role, as it forms–together with the CD18 molecule–LFA-1 (lymphocyte function-associated antigen 1), interacting with intercellular adhesion molecule 1 (ICAM-1) located, inter alia, on endothelial cells. Integrin alpha M (ITGAM, CD11b) is a molecule responsible for leukocyte adhesion to endothelial cells, and when combined into a complex with the CD18 molecule, also stimulates the migration of these cells through the wall of the vessel. Another integrin is ITGAX (integrin alpha X) or CD11c. The innate immune system is regulated, among others, by changes in the expression of these molecules on the surface of immunocompetent cells [9].

Toll-like receptors (TLRs) are transmembrane and intracellular proteins, that recognize exogenous and endogenous, structurally conserved, molecules. TLR-2, often simultaneously with TLR-1 and TLR-6, can detect the largest number of pathogen-associated molecular patterns (PAMPs) and several endogenous deteriorations or danger-associated molecular patterns (DAMPs), such as heat shock proteins HSP-60, HSP-70, HMGB-1 and gp96 [10]. TLR-4 recognizes Gram-negative lipopolysaccharides (LPS) and viral proteins, as well as endogenous DAMPs: oxidized low-density lipoproteins, biglycan, fibrinogen, beta-amyloid, hyaluronate and the aforementioned HSPs [11]. To this day, there have been no published literature data reporting alterations of TLR expression in children with FH treated using statins.

So far, the evidence of the role of T and B lymphocytes in hypercholesterolemia-induced atherosclerosis has been derived mainly from animal models only. There are few studies on lymphocytes in patients with hypercholesterolemia, but only one report concerning the blood lymphocyte subpopulations in children with FH has been published [12]. Noticeably, there were no such studies conducted on children treated with statins.

Children with FH, though without comorbidities or other risk factors, may be an excellent model of early-stage, preclinical atherosclerosis. Studies within this age group might show the changes in blood vessels, i.e., when the inflammation, still secondary to the accumulation of modified lipids, is less severe compared with symptomatic adults.

Taking into account the pleiotropic effects of statins and the sensitivity of the developing immune system in children, the present study aimed to assess the influence of statin treatment in children with familial hypercholesterolemia on parameters of the cellular immunity. Our work was designed to address the following questions: (i) Are there differences between children with FH, treated with simvastatin vs. non-treated, in the amounts of individual types of white blood cells assessed in peripheral blood smears? (ii) Is the expression of adhesion molecules on granulocytes and TLR-2 and TLR-4 on granulocytes, lymphocytes and monocytes different in children with FH treated with statins compared to those treated only with a specific dietary regimen? (iii) Does the distribution of basic subpopulations of blood lymphocytes of children with FH differ depending on the treatment modality used, i.e., statins or dietary?

## 2. Materials and Methods

### 2.1. Study Participants

This study was designed as cross-sectional research. Out of all individuals followed in the Metabolic Outpatient Clinic at the Bialystok University Children’s Hospital, 13 children with FH were included, in whom dietary low-cholesterol regimen and 10 mg simvastatin once daily for a minimum of 26 weeks had been used. Thirteen children with FH, matched by age and gender, were enrolled in the control group. Those participants were treated exclusively with the low-cholesterol diet for at least 26 weeks before their inclusion in the study, whereas they had never been treated with statins. All studied parameters were assessed only once, i.e., at a single time point. During the study, children did not report any complaints, did not take any other medication and did not have clinical symptoms of acute infection either, based on the concentration of C-reactive protein which remained within the normal range. Exclusion criteria were secondary causes of hypercholesterolemia, and the age under 10 years (according to the official age-specific registration approval for simvastatin). The FH diagnosis was based on molecular tests and/or clinical criteria [13]. All participants underwent anthropometric measurements, routine physical examination, blood pressure examination using standard methods and a fasted glucose level. The limited sample size reflected a rare prevalence of this congenital disease, and also resulted from strict inclusion criteria applied in this study. Matching the control group for age and sex reduced potential confounding effects. Missing data associated with analytical issues were not replaced by conjectural values. The detailed characteristics of the study groups are shown in Table 1.

This study was performed in line with the principles of the Declaration of Helsinki and was approved by the institutional ethics committee (R-I-002/389/2014 and R-I-002/388/2014). Written informed consent was obtained from parents/legal guardians. The data underlying this article are available in the article and in its online Supplementary Materials.

### 2.2. Methods

#### 2.2.1. Hematological and Biochemical Measurements

A sampling of fasted venous blood was conducted in the early morning after the whole-night fasting. Hematological laboratory parameters were immediately measured on a daily basis. Total WBC and differential counts were determined using EDTA whole blood and were later quantified using an automated hematology analyzer S-XT41 (Sysmex, Kobe, Japan). Glucose was determined using the enzymatic hexokinase/glucose 6-phosphate dehydrogenase method (COBAS 6000 C501, Roche Diagnostics, Mannheim, Germany). CRP was assessed using the immunoturbidimetric method (COBAS 6000 C501, Roche Diagnostics, Mannheim, Germany). Total cholesterol, LDL- and HDL-cholesterol and triglycerides were measured with the colorimetric enzymatic method (COBAS 6000 C501, Roche Diagnostics, Mannheim, Germany).

#### 2.2.2. Flow Cytometry Assessment

Leukocytes were prepared using a whole blood lyse no-wash method, according to the manufacturer’s recommendations (Becton Dickinson). Preparations were analyzed on an 8-color flow cytometer (FACSCanto™ II, Becton Dickinson, Franklin Lakes, NJ, USA), using routine methods. In brief, the main leukocyte subsets (neutrophils, monocytes, lymphocytes) were identified by forward vs. side-scatter assessment. To validate the separation of lymphocytes, CD45 was used, whereby CD14 expression was used to further validate the separation of monocytes and neutrophils.

Within the lymphocyte compartment CD3, CD4, CD8 and CD19 surface markers were used to identify main lymphocyte subsets (cytotoxic T cells, helper T cells and B cells). Mean fluorescence intensity (MFI) of the lymphocytes and monocytes compartments was assessed using the expression of the surface markers CD11a, CD11b, CD11c, CD18, CD45-RA, CD45-RO, HLA-DR, CD-69, CD282 (TLR2) and CD284 (TLR4) on evaluated cells. All fluorescent-labeled antibodies were purchased from Becton Dickinson Life Sciences (Warsaw, Poland), except CD282 and CD284, which were obtained from Beckman Coulter (Warsaw, Poland).

#### 2.2.3. Statistical Analysis

The percentage of receptor-bearing cells and the absolute cell numbers for a given surface molecule were calculated. For quantitative variables, the arithmetic mean and standard deviation (SD) were calculated. The variable distribution was evaluated using the Kolmogorov–Smirnov test. Because the tested variables were inconsistent with normal distribution, a Mann–Whitney two-sided rank U-test was used. The differences between the studied values were considered statistically significant at a *p*-level < 0.05.

The statistical analyses were performed with the STATISTICA software (version 12, Tibco Software Inc., Palo Alto, CA, USA).

## 3. Results

No significant differences in anthropometric traits were found between the studied groups due to the adjustment methods used and pattern matching for age and sex. In the group treated with statins, the molecular diagnosis of FH was confirmed in 10 children (77%), whereas in those treated without statins, 8 children had been diagnosed using a molecular test (62%). In subjects treated with the diet and statins, significantly lower cholesterol levels and lower cholesterol lipoprotein LDL were found in comparison to those without statins. The impact of the pharmacological therapy and the range of cholesterol reduction was consistent with the data on the effectiveness of statins in children reported elsewhere [7].

### 3.1. Blood Count

No significant differences were found in basic values of peripheral blood count between the examined groups of children with hypercholesterolemia (Table 2). They did not differ in the number of erythrocytes, hemoglobin concentration, hematocrit and platelets. The leukocyte count was comparable in both groups. In addition, the distribution of individual types of white blood cells did not differ significantly between the group of children treated with statins and those treated only with diet and lifestyle modification. Neither neutropenia nor lymphopenia was found in the examined participants.

The ratio of granulocytes to lymphocytes in both studied groups did not differ significantly and was 1.54 ± 0.7 in the group treated with statins, and 1.68 ± 0.51 in the group treated only with the diet.

### 3.2. Adhesive Molecules and TLR Expression

**Figure 1:**
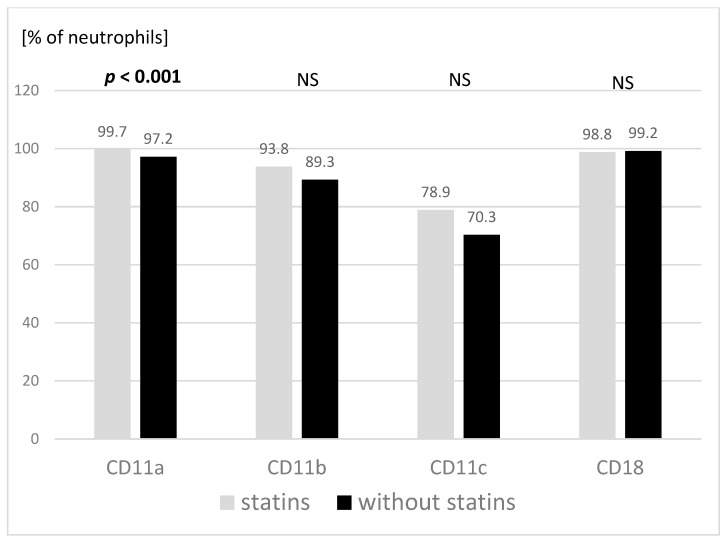
Expression of adhesion molecules on granulocytes in the studied groups with respect to statin treatment (treated with simvastatin *n* = 11; not treated with simvastatin *n* = 12). Mann–Whitney two-sided rank U-test was used. NS: not significant.

The percentage of granulocytes showing expression of the CD11a molecule on their surface was significantly higher in the group treated with statins. Similar, though not significant, relationships were observed in the expression of the CD11b and CD11c molecules (Figure 1). The groups did not differ in the proportion of granulocytes showing expression of the CD18 molecules.

**Figure 2:**
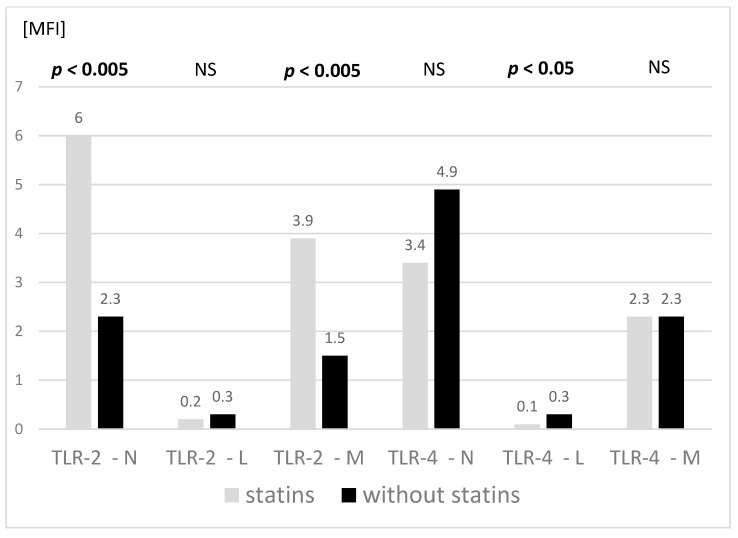
Expression of TLR-2 and TLR-4 on peripheral blood cells in the examined groups (N–neutrophils, L–lymphocytes, M–monocytes). Treated with simvastatin *n* = 11, not treated with simvastatin *n* = 9. Mann–Whitney two-sided rank U-test was used. NS: not significant. MFI: Mean fluorescence intensity.

The TLR-2 expression analysis on different peripheral blood cells showed significantly higher values on granulocytes and monocytes of statin-treated children, with no differences in the expression of these molecules on lymphocytes. Furthermore, the expression of TLR-4 was lower in this group on lymphocytes (significantly) and on granulocytes (trend). No differences were found in the TLR-4 expression on monocytes between the tested groups (Figure 2).

### 3.3. Main Subpopulations of Peripheral Blood Lymphocytes

**Figure 3:**
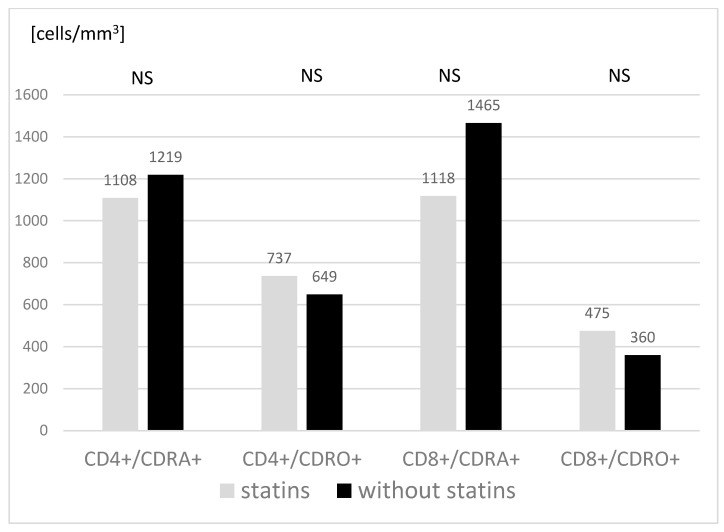
Expression of RA and RO maturation markers on T lymphocytes in studied subjects. Treated with simvastatin *n* = 11, not treated with simvastatin *n* = 12. Mann–Whitney two-sided rank U-test was used. NS: not significant.

The evaluation of the basic subpopulations of peripheral blood lymphocytes did not show significant differences between the study groups either (Table 3). The percentages and absolute values of T lymphocytes (CD3+), helper T lymphocytes (CD4+), cytotoxic T lymphocytes (CD8+) and B lymphocytes (CD19+) remained within the age-matched reference range in all children, and did not differ significantly between the two groups concerning the statin use (treated vs. not treated). There were no significant determinants of acute or chronic activation (defined by expression of HLA-DR and CD69) of either helper or cytotoxic T lymphocytes. The absolute and percentage values, and the relative proportions of naive (CD45-RA) and memory (CD45-RO) lymphocytes did not significantly differ in either the helper or cytotoxic T lymphocytes subpopulations between the studied groups. The graphic presentation of the obtained results indicates a tendency to lower the percentage of naive cells (CD45-RA) and a higher percentage of memory cells (CD45-RO) in children with hypercholesterolemia treated with statins, particularly regarding the cytotoxic lymphocyte subpopulation (CD8+) (Figure 3).

## 4. Discussion

In FH children treated with a low-cholesterol diet and statins, significantly lower total cholesterol levels and lower cholesterol lipoprotein LDL were found, compared with the controls without statins. Thus, statins could be effective in pediatric patients with this rare condition. Furthermore, our findings were consistent with other studies reporting a similar response to statin treatment in children [7]. In the present study, the simvastatin therapy in children with FH was not associated with alterations in the number of granulocytes and monocytes. There was no association between statin treatment and the pattern of peripheral blood lymphocyte subpopulations either. The percentage of granulocytes with the expression of adhesion molecules was higher in those treated with statins. Moreover, the TLR-2 expression on granulocytes and monocytes showed higher values, whereas the TLR-4 expression on lymphocytes and granulocytes was lower in simvastatin-treated children than in the controls without treatment.

Studies in adults have shown that an increased number of leukocytes is a strong and independent risk factor for atherosclerosis [14]. Among white blood cells, an increased amount of granulocytes is a risk factor for the unfavorable course of ischemic heart disease [15]. It has been also shown that an increased value of neutrophil-lymphocyte ratio (NRL) in adults is related to the worse outcomes of ischemic heart disease [16] and ischemic stroke [17]. On the other hand, statins have been proven to reduce NLR [18]. It has been estimated that in patients with stable ischemic heart disease, a 6-month-long therapy with pravastatin decreases the granulocyte count by 9% [19]. A large Danish study in adults demonstrated that the statin treatment was associated with more monocytes and lymphocytes in the peripheral blood, with an unaffected granulocyte count [20].

In the literature, there are incidental reports available evaluating, simultaneously, the cholesterol concentration and white blood cell count in children. An Italian report published in 2001, encompassing 1171 children aged 11 years, showed an essential direct relationship between leukocytosis and total cholesterol concentrations [21]. A study, comparing the smears of 23 children with FH with healthy controls, showed no differences except for a higher percentage of monocytes [12]. The study conducted by Tolani et al. was performed on 50 children with FH and showed more monocytes in the smear of children with lower HDL cholesterol concentrations, but failed to detect an association with the LDL cholesterol [22].

In our study, no differences in the total amount of leukocytes were found between the children with FH: those treated with statins vs. not treated. No differences were found regarding the number of granulocytes, lymphocytes or monocytes either. Furthermore, there was no significant difference in the ratio of granulocytes to lymphocytes (NLR) between the studied groups.

If no other risk factors are present, atherosclerosis during growth and development remains subclinical, even in patients with heterozygous FH. The process of atherogenesis begins with an increased adhesion of the leukocytes to endothelial cells. This interaction largely depends on the expression of adhesion molecules on the immune cells. Adult studies indicate a higher expression of the CD11b and CD18 molecules in patients with ischemic heart disease [23], but also in those with “pure” hypercholesterolemia without complications [24]. The treatment with simvastatin at different doses has been shown to reduce the expression of adhesive molecules on monocytes among adults with symptomatic atherosclerosis [25]; however, some other studies did not support such an effect in adults with FH [26]. These reports concerned the expression of CD11b and CD18 on monocytes, whereas only one of them focused on the expression of different molecules on granulocytes, monocytes and lymphocytes, just as in our study. Stulc et al. demonstrated a higher expression of CD11b and CD18 on all the cell types in hypercholesterolemic adults compared with healthy ones, but found no differences in CD11a expression.

Thus far, only one published study dealt with an adhesion molecule peripheral blood cells’ expression in children with FH [27], reporting that the expression of the CD11b and CD18 molecules on monocytes of children with FH was lower than on monocytes of healthy peers. In our study, the expression of adhesion molecules on granulocytes did not differ between the groups with FH, except for a slightly higher expression of CD11a in the group treated with statins.

The hypolipemic action of statins itself should be associated with a reduction in the number of adhesion molecules, because a reduction in the expression of adhesion molecules on leukocytes of patients with a homozygotic form of FH after LDL apheresis has been demonstrated [28]. However, studies from animal models indicate that inconsistent or contrasting results may also stem from other actions of statins, e.g., the inhibition of isoprenoid production and secondary inhibition of intracellular transmission. Statins reduce the activation of endothelial cells and influence redox processes. So, each of these pathways may trigger compensatory actions that balance the changes resulting from direct hypolipemic effects [11]. Further, even a lower expression of CD11a on leukocytes could be found in individuals treated with statins compared with healthy controls [29]. Several compensatory mechanisms are particularly effective during the development period.

The role of TLRs in the development of atherosclerosis has been extensively studied, but most reports are concerned with in vitro and animal models [30]. The intracellular stimulation of TLRs affects—through the activation of the NFkB transcription factor pathway—the expression of cytokines, adhesion molecules, the maturation of dendritic cells and the readiness of T lymphocytes for a specific response [31]. When stimulated by PAMPs, it is protective against microorganisms, while DAMPs trigger “sterile” inflammation involved in tissue damage repair, but also play a pathogenic role in autoimmune diseases, atherosclerosis and cancer [10].

Treatment with statins leads to a decreased activity of the NF-kB transcription factor and therewith an associated reduction in the synthesis of cytokines, chemokines, adhesion molecules and TLRs [32], thus, inhibiting the inflammatory reaction. It is still debatable whether this mechanism is related to the reduction of isoprenyl derivatives of mevalonic acid [33], the accumulation of mevalonic acid or whether it is indirectly associated with the effect of statins on lipid metabolism [32]. Some recent well-designed proteomic studies, based on a modern bioinformatics analysis, have also demonstrated a novel, though not yet known, mechanism of these interactions [34].

Moutzouri et al. showed a higher expression of TLR2 and TLR4 on monocytes in adult patients with hypercholesterolemia than in the controls. After three months of treatment with high-dose simvastatin (*n* = 30) or low-dose simvastatin combined with ezetimibe (*n* = 30), the expression of these receptors decreased to the values observed in the healthy controls. This well-designed study showed that higher values were observed in the adults with hypercholesterolemia, but without typical atherosclerosis symptoms. It is worth noting that the study participants were also treated with a low-fat and low-cholesterol diet [35]. This is important in the context of some reports on the possible impact of a fat-rich, high-energy diet on the expression of TLR2 and TLR4 [36]. However, another relevant study has not demonstrated the effect of statins on TLR4 expression [37]. The data on statin’s effect on the expression and activation of TLR2 are scarce, and studies regarding this receptor are also inconclusive [32], but still, there are no such reports in children with FH. The impact of statin treatment in this age group was not assessed either, according to our knowledge.

In our study, all participants were treated with a combined regimen of a low-cholesterol and low-fat diet. Unlike in the study of Moutzouri et al. we observed higher values of TLR2 expression on granulocytes and monocytes in FH children treated with statins compared to those not treated. In terms of the TLR4 expression on granulocytes, we found lower, though not significant, values on granulocytes, significantly lower on lymphocytes and, contrary to the above study, no differences in the TLR4 expression on monocytes of children treated with statins.

There are no comparable data on the TLR expression on granulocytes and lymphocytes, either in children or adults, and results on monocytes in adults with hypercholesterolemia are conflicting.

Muldoon et al. found higher absolute amounts of total lymphocytes, T helper lymphocytes and cytotoxic T lymphocytes in men with higher cholesterol levels compared to those with normal cholesterol [38]. Another study by the same research team showed no significant differences in lymphocyte subpopulations in adults following 6 months of lovastatin treatment [39]. Collado et al. evaluated lymphocyte subpopulations in adults with hypercholesterolemia, and showed a significantly lower amount of T helper lymphocytes in comparison to healthy controls, but no differences in total T and T cytotoxic lymphocytes. The significantly higher expression of the CD69 molecule (early activation marker) was shown in T lymphocytes, and also separately in helper and cytotoxic T lymphocytes [40].

There are only limited data assessing immune cells in children with hypercholesterolemia. Among children undergoing 6 months of dietary treatment, the decrease in the absolute number of T lymphocytes and subpopulations CD4+ and CD8+ was observed [41]. The only study available to specifically compare lymphocyte subpopulations in children with hypercholesterolemia and healthy children showed no differences [12]. We did not find such differences either between children with FH treated only with the diet and children on both diet and statins. Furthermore, we found no differences in the number of T lymphocytes, showing early (CD69) and chronic activation (HLA-DR) markers on their surfaces.

A study by Christensen et al. showed a lower percentage of naive T lymphocytes (CD4+ CDRA+) in healthy children than in their peers with hypercholesterolemia [12]. A similar pattern in the children treated with statins compared to those treated only dietetically was observed in our study for both main T lymphocyte subpopulations (CD4+ and CD8+). Even if statistical significance failed for the differences, the trend could be of interest.

A meta-analysis of 4655 adult patients treated with statins and placebo-controlled failed to show any impact of the active treatment on the incidence of infections or mortality associated with infections [42]; nevertheless, there have been no such data available for the pediatric population. Pediatric FH patients must be treated in a different and perhaps a more cautious manner compared with adult patients, as the therapy with statins begins in childhood even before the onset of complications and exacerbation of inflammatory processes associated with atherosclerosis. In general, these medicines have not been used in the pediatric population for a long time; however, the statin-related moderate immunomodulatory and anti-inflammatory effect, implemented reasonably early, can presumably provide certain preventive measures. Knowledge of the effects of statins during growth and development, particularly interaction with the immature immune system, will become important if upcoming guidelines demonstrate that adolescent FH patients require stronger anti-inflammatory drugs to further reduce the future risk of atherosclerosis.

A limitation of our study is its observational nature. Due to the nature of the rare disease, the number of participants was small. Familial hypercholesterolemia in children is being still underdiagnosed and underestimated, and more importantly, appropriate pharmacological therapies are unfortunately implemented even more rarely. We had a limited ability to control the disruptions that were associated with some children remaining in care for extended periods; thus, some individuals may have dropped out from care early. Furthermore, an observational study has essential limitations regarding the cause-and-effect relationship. However, there was no possibility to conduct a placebo-controlled trial due to ethical issues, given that a recommended and effective treatment was fully available. Therefore, children with FH on simvastatin therapy were compared with their non-treated peers whose parents refused to consent to pharmacological treatment. The strength of our study was a rigorous protocol based on strict inclusion and exclusion criteria using precise age- and sex-matching. Further, a relatively long treatment duration in this study may have reassured reliability. There have been only very few data available regarding the safety of statin treatment in children, while published studies in adults with FH were usually limited to a period of two months or shorter.

In conclusion, our data show that long-term treatment with statins in children with FH is related to a significant reduction of cholesterol levels, and does not lead to alterations in the number of granulocytes and monocytes, which are essential cellular components of non-specific immunity. We extend our observation by demonstrating that there is no association between treatment with statins and the number or distribution of peripheral blood lymphocyte subpopulations in children with FH. The differences in the expression of adhesion molecules and TLRs on the surface of immunocompetent cells need to be re-examined, because the influence of statins on the innate immune system may be critical for their effectiveness and safety during growth and development.

## Figures and Tables

**Table 1:** Characteristics of studied children with familial hypercholesterolemia treated with statins and the diet or treated with an exclusive low-cholesterol diet for 6 months. Data are presented as means and standard deviations where applicable. A Mann–Whitney two-sided rank U-test was used.

	Statins *n* = 13	Without Statins *n* = 13	*p*
Age [years]	14.0 (1.9)	13.6 (1.9)	0.511
Sex (Girls/Boys)	9/4	9/4	
Mutation status			
–LDL-R	7 (54%)	5 (38%)	
–APO-B	3 (23%)	3 (23%)	
–neither LDL nor APO-B	1 (8%)	1 (8%)	
–not performed	2 (15%)	4 (31%)	
Weight [kg]	54.1 (12.58)	57.1 (13.62)	0.488
Height [m]	1.6 (0.09)	1.6 (0.08)	0.840
Systolic blood pressure [mmHg]	116.2 (9.47)	105.9 (30.44)	0.347
Diastolic blood pressure [mmHg]	72.1 (9.79)	71.8 (9.37)	0.852
Heart rate [bpm]	80.4 (16.65)	84.2 (14.5)	0.458
Glucose [mg/dL]	87.3 (8.07)	94.8 (9.05)	0.014
Total cholesterol [mg/dL]	220.8 (28.81)	257.4 (26.84)	0.003
HDL cholesterol [mg/dL]	63.5 (18.58)	56.4 (12.96)	0.418
LDL cholesterol [mg/dL]	143.9 (25.68)	186.1 (26.89)	0.001
Triglyceride [mg/dL]	65.6 (18.35)	74.8 (23.86)	0.362

**Table 2:** Blood count and differential count of major leukocyte groups in children with familial hypercholesterolemia treated with a combination of statins and a low-cholesterol diet, and those on an exclusive low-cholesterol diet for 6 months. The data are shown as mean values and standard deviation (in brackets). A Mann–Whitney two-sided rank U-test was used.

	Statins *n* = 13	Without Statins *n* = 13	*p*
RBC [10^6^/µL]	4.8 (0.28)	4.9 (0.31)	0.657
HGB [g/dL]	13.4 (0.48)	13.4 (0.67)	0.909
HCT [%]	39.8 (1.48)	40.2 (1.98)	0.392
PLT [10^3^/µL]	286.6 (47.72)	256.9 (44.51)	0.101
WBC [10^3^/µL]	5.8 (2.00)	6.0 (1.24)	0.186
Neutrophils [10^3^/µL]	3.0 (1.64)	3.2 (0.90)	0.376
Lymphocytes [10^3^/µL]	1.9 (0.40)	2.0 (0.43)	0.979
Monocytes [10^3^/µL]	0.5 (0.16)	0.5 (0.11)	0.810
Eosinophils [10^3^/µL]	0.2 (0.18)	0.2 (0.26)	0.531
Basophils [10^3^/µL]	0.1 (0.04)	0.1 (0.06)	0.733

**Table 3:** Main subpopulations of peripheral blood lymphocytes in studied participants. The data are presented as a percentage of receptor-bearing cells and the absolute cell numbers. Mean values and standard deviations (in brackets) are shown. Mann–Whitney two-sided rank U-test was used.

		Statins *n* = 13	Without Statins *n* = 12	*p*
T cells (CD3+)	%	69.8 (4.78)	68.6 (4.26)	0.469
	cells/µL	1357.8 (338.74)	1365.9 (367.77)	0.909
T helper cells (CD3+/CD4+)	%	39.8 (5.68)	39.8 (6.14)	0.894
	cells/µL	770.0 (210.59)	780.2 (212.85)	0.820
T helper late activated CD3+/CD4+/HLADR+	%	3.5 (1.54)	3.3 (1.34)	0.936
	cells/µL	67.9 (32.07)	69.5 (34.06)	0.865
T helper early activated CD3+/CD4+/CD69+	%	0.5 (0.61)	0.5 (0.51)	0.887
	cells/µL	8.8 (11.37)	11.3 (13.45)	0.821
T cytotoxic cells (CD3+/CD8+)	%	30.3 (5.68)	29.3 (6.48)	0.574
	cells/µL	556.0 (191.99)	597.7 (241.71)	0.865
T cytotoxic late activated CD3+/CD8+/HLADR+	%	4.2 (2.33)	4.4 (2.74)	0.728
	cells/µL	80.8 (49.51)	83.6 (49.58)	1.000
T cytotoxic early activated CD3+/CD8+/CD69+	%	1.1 (1.28)	0.5 (0.66)	0.190
	cells/µL	21.4 (29.59)	10.3 (10.88)	0.525
B cells (CD19+)	%	15.0 (4.84)	18.8 (7.41)	0.152
	cells/µL	275.6 (139.33)	348.9 (163.62)	0.392

## Data Availability

The data underlying this article are available in the article and in its online Supplementary Materials.

## Supplementary Materials

The following supporting information can be downloaded at: https://www.mdpi.com/article/10.3390/jcm11102924/s1. Raw data used in the publication.
